# Human Metapneumovirus G Protein Immunogenicity and Safety Explored via Carrier Protein Fusion

**DOI:** 10.3390/tropicalmed11050135

**Published:** 2026-05-15

**Authors:** Tian Ren, Kailun Ma, Xinmiao Lai, Jizheng Chen, Changgui Li

**Affiliations:** 1 State Key Laboratory of Respiratory Disease, National Clinical Research Center for Respiratory Disease, Guangzhou Institute of Respiratory Health, The First Affiliated Hospital of Guangzhou Medical University, Guangzhou 510120, China; ren_tian@gzlab.ac.cn; 2 Guangzhou National Laboratory, No. 9 XingDaoHuanBei Road, Guangzhou International Bio Island, Guangzhou 510005, China; 3 National Institutes for Food and Drug Control, Beijing 102629, China

**Keywords:** HMPV G, carrier protein, CTB, immunogenicity, pulmonary inflammation, antiviral effect

## Abstract

Human metapneumovirus (HPMV) is a significant pathogen that causes lower respiratory tract infections. Given the weak immunogenicity thereof, and the few relevant studies, the utility of the viral membrane protein G as a vaccine remains controversial. In this study, the G extracellular domain (RMG) of HMPV was expressed either alone or fused with the cholera toxin B subunit (CTB) and “cross-reacting material 197” (CRM197) carrier proteins (giving G-CTB/G and CRM197), to enhance immunogenicity. The non-glycosylated G protein (REG) expressed in *Escherichia coli* served as a control. SDS-PAGE and anti-His tag Western blotting verified that each protein was successfully expressed and correctly identified. BALB/c mice were immunized with each protein and subjected to challenge with HMPV. The results showed that, although immunization with RMG alone failed to induce potent neutralizing antibodies, it modestly reduced viral loads in the lungs of mice. However, the pathological damage caused by lung inflammation was more aggravated than that of the control challenge group. The level of specific IgG antibody induced by the recombinant G-CTB was significantly higher than that elicited by RMG. Compared to the RMG group, the viral load in the lungs of the G-CTB group tended to be reduced. Also, the damage caused by lung inflammation was significantly alleviated. Our study proves that HMPV G may be a valuable antigen in terms of HMPV vaccine development and offers a promising strategy for modulating the immunogenicity and safety thereof.

## 1. Introduction

Human metapneumovirus (HMPV) is a negative-stranded RNA virus of the family Parapneumoviridae that was first isolated from respiratory tract samples of Dutch children in 2001 [1,2,3]. In the time since its discovery, HMPV has emerged as one of the primary pathogens causing respiratory infections worldwide [4]. Globally, HMPV is responsible for 5–7% of all respiratory infections in children admitted to hospitals. In general populations seeking medical advice, HMPV accounts for about 3% of all respiratory tract infections [5].

The clinical manifestations of HMPV infection vary widely, ranging from symptoms similar to those of the common cold to severe respiratory diseases, such as bronchiolitis or pneumonia [6,7]. This poses particularly serious threats to older adults, immunocompromised individuals, and patients with underlying chronic diseases, in whom severe infections can be fatal [8,9]. Despite the increasing clinical impact of HMPV, therapeutic agents and vaccines remain in early stages of development. No specific treatment or vaccine is currently authorized [7,10,11].

HMPV features two subtypes, A and B [12]. The genome codes for nine proteins, including three surface transmembrane proteins: a glycoprotein (G), a small hydrophobic protein (SH), and a fusion protein (F) [13]. Protein G is a type II transmembrane protein, the N-terminus of which resides in the cytosol, whereas the C-terminus is located extracellularly [4]. Although it has been reported that direct immunization with protein G does not elicit a protective response, or only a weak response, protein G may facilitate virus attachment during infection, thereby inhibiting the production of important immune and antiviral mediators such as chemokines and type I interferon, as has been proven by deleting the G protein-encoding gene from the full-length genome [14]. Accordingly, a recombinant HMPV lacking the G protein (rhMPV-ΔG) was developed as a potential vaccine candidate and was both attenuated and immunogenic in a rodent model of infection [15]. However, a later study by Dubois and colleagues found that a recombinant strain of a patient-derived virus from which the SH-encoding gene had been deleted afforded better attenuation and protection in mice than did the virus lacking the G protein [16]. In 2022, Velayutham and colleagues studied the role played by the G protein in terms of modulating the immune response, and reported that, although rhMPV-ΔG immunization conferred some protection in mice, the animals exhibited a number of signs indicating that lung disease was enhanced at the earliest time points after challenge compared to the pathology of mice primarily infected with the rhMPV-WT strain [13]. Together, the data indicate that G protein should still be considered as a possible vaccine, as an immune response might block or inhibit the modulating effect of the protein during an actual infection.

We immunized BALB/c mice with the extracellular domain of the HMPV G protein. However, in view of the poor immunogenicity thereof and the intrinsic glycosylation characteristics [15,17,18], we fused the G extracellular domain with two carrier proteins—the cholera toxin B subunit (CTB) [19] and “cross-reacting material 197” (CRM197) [20], respectively. Both fusion proteins markedly enhanced G protein immunogenicity, and fusion to CTB avoided pathological aggravation during virus challenge. Our study proves that HMPV G protein may be a useful target antigen for HPMV vaccine development, and offers a promising strategy for modulation of the immunogenicity and safety thereof.

## 2. Materials and Methods

### 2.1. Animals

Specific pathogen-free (SPF) 6–8-week-old female BALB/c mice were supplied by the Laboratory Animal Center of the Guangzhou National Laboratory. All mice had been procured from Guangdong Vital River Laboratory Animal Technologies Co., Ltd. (Foshan, Guangdong, China; SCXK [Yue] 2022-0063). This study was approved by the Guangzhou National Laboratory Animal Ethics Committee on 17 December 2024 (approval number GZLAB-AUCP-2024-10-A10).

### 2.2. Mouse Immunization and Challenge

The mice were randomly divided into five groups (*n* = 8 per group) and some were immunized intramuscularly via injection into the bilateral quadriceps femoris on days 0, 21, and 42. The immunization regimen was based on those of published vaccine studies. Proteins were quantified using the bicinchoninic acid assay, diluted to 0.5 mg/mL with PBS, and administered at 50 µg per mouse per dose. Control mice received an equal volume of sterile PBS.

Blood samples were collected 14 days after the third immunization (day 56). On that day, mice were intranasally challenged with 1 × 10^6^ plaque-forming units (pfu) of HMPV. Five days post-challenge (day 61), the mice were anesthetized, and lung tissues were harvested for viral load determination and histopathological analysis.

### 2.3. Detection of Viral Loads in Mouse Lungs

The right lung lobe was harvested, weighed, submerged in TRIzol, and homogenized for 3 min in an automated tissue homogenizer (TissueLyser II, QIAGEN, Hilden, Germany) that contained steel balls. Homogenization was paused for 10 s every minute. The samples were centrifuged at 12,000 rpm for 15 min at 4 °C; 200 µL aliquots of each supernatant were collected, and these were added to 0.8 mL of a TRIzol-containing solution prior to total RNA extraction. Quantitative real-time PCR (qPCR) was performed to measure viral genomic RNA copy number, a widely accepted indicator of viral burden in HMPV infection models. The HMPV F gene was targeted using the following specific primers:

Forward: 5′-GCCGTTTCTAACATGCCGAC-3′

Reverse: 5′-CCCTACTCTGTTGCTGCCAA-3′

Quantitative real-time PCR (qPCR) was performed using a defined concentration of the empty pcDNA3.1(+) plasmid as the internal reference standard. Each sample was analyzed in triplicate, and viral titers were calculated from the corresponding standard curve.

### 2.4. Pathological Examination of Mouse Lungs

The left lung lobe was immediately fixed in 4% formalin and sent to Servicebio Co., Ltd. (Wuhan, Hubei, China) for paraffin embedding, sectioning, and hematoxylin-and-eosin (H&E) staining. After scanning, histopathological lesions were comprehensively evaluated and scored using a semi-quantitative system [21].

Seven pathological indicators were each scored from 0 to 3, with the total score ranging from 0 to 21; higher scores reflected more severe inflammatory injury. Scoring was performed independently by two blinded observers, and the final score was the mean of the two evaluations. The detailed scoring criteria are as follows (Table 1):

**Table 1:** Scoring criteria for histopathological lesions in mouse lung tissues.

Score	Bronchiolar/Bronchial infiltrate Area (%)	Bronchiolar/Bronchial Infiltrate Morphology	Luminal Exudate	Perivascular Infiltration	Alveolitis	Hemorrhage	Interstitial Pneumonia
0	None	None (rare mild infiltrates or peribronchial lymphoid aggregates, consistent with normal animals)	None	None	None	None	None
1	Slight (<25%)	Mild, abnormal	<10%	<10%	Mild: thickened alveolar walls with preserved architecture	Hemorrhage area <10%	Mild (lesion area <25% of total lung)
2	Moderate (25–75%)	Intermittent circumferential or crescent-shaped infiltrates, thickness <5 cells	10–25%	10–50%	Moderate:expanded alveolitis, occasional inflammatory cells extending into the lumen	Hemorrhage area 10–50%	Moderate (lesion area 25–50% of total lung)
3	Severe (>75%)	Severe circumferential infiltrates, thickness >5–10 cells	≥25%	>50%	Severe: abundant inflammatory cells with honeycomb appearance	Hemorrhage area >50%	Severe (lesion area >50% of total lung)

### 2.5. Extracellular Domains of G Proteins and Recombinant G Protein Constructs

**Figure 1:**
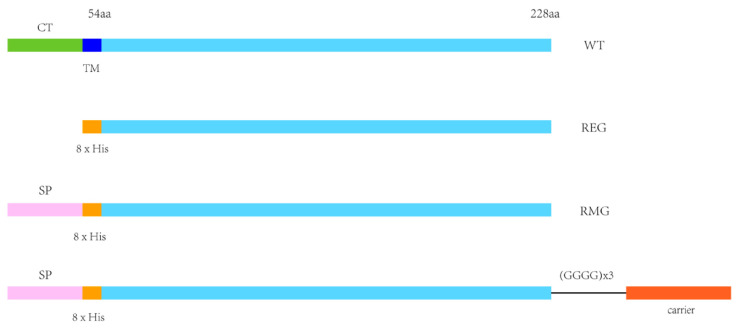
Schematic representation of the HMPV G protein and the recombinant constructs. The key structural domains and tag annotations are as follows: CT (cytoplasmic tail, green segment); TM (transmembrane region, dark blue segment); extracellular region (light blue segment); SP (signal peptide, mouse Ig heavy chain, MGWSCIILFLVATATGVHS, pink segment); 8×His (orange segment); (GGGGS)_3_ (flexible linker sequence, black straight line); polysaccharide-binding vaccine carrier (CTB/CRM197/DT, red segment). WT: Full-length wild-type HMPV G protein; REG: Prokaryotically expressed G protein (extracellular domain only, with the 8×His tag); RMG: Eukaryotically expressed G protein (with the signal peptide and 8×His tag); G-carrier protein: Eukaryotically expressed G protein linked to the polysaccharide-conjugated vaccine carrier via the (GGGGS)_3_ linker.

The extracellular domain of the G protein was derived from the HMPV B1 strain. When preparing the prokaryotic REG construct, the cytoplasmic and transmembrane domains were deleted, leaving only the extracellular domain. An 8×His tag was fused to the N-terminus to facilitate solubility, and expression in and purification from *Escherichia coli*. Codon-optimized gene fragments were synthesized and cloned into PET30a (+) (Figure 1).

To prepare the eukaryotic RMG, G-DT, G-CRM197, and G-CTB constructs, a mouse immunoglobulin heavy-chain signal peptide (SP: MGWSCIILFLVATATGVHS) was added to the N-termini to drive protein secretion into the culture supernatant. An 8×His tag was also fused to each N-terminus to enable affinity-based purification. The HMPV G ectodomain and each carrier protein were connected by a (GGGGS)_3_ linker that ensured structural flexibility. All required gene fragments were cloned into pcDNA3.1(+) (Figure 1). The amino acid sequences and NCBI RefSeq accession numbers are listed in Supplementary Table S1.

### 2.6. Cells and Viruses

HMPV AY297749 (subtype A) or AY525843 (subtype B) was provided by Sinovac Biotech Ltd. (Beijing, China). Vero E6 cells were cultured in Dulbecco’s modified Eagle’s medium (DMEM) supplemented with 10% fetal bovine serum, 1% penicillin–streptomycin, and 1% L-glutamine. At 90% confluence, cells were digested with 0.25% EDTA-trypsin and seeded into T75 flasks at 1 × 10^5^ cells/mL. Recombinant HMPV-GFP was inoculated at a multiplicity of infection (MOI) of 0.1 together with 10 μg/mL TPCK-trypsin, and the cells were then incubated at 37 °C under 5% CO_2_. After 6–7 days, the cell–supernatant mixture was freeze-thawed twice at −80 °C and centrifuged at 5000 rpm for 10 min at 4 °C. The supernatant was collected, concentrated with PEG8000, and resuspended in DMEM to prepare the virus stock.

Viral titers were determined in 96-well plates via 10-fold serial dilutions. After 2 days of incubation, cytopathic effects were observed, and TCID_50_ values were calculated using the Reed–Muench method.

### 2.7. Protein Expression and Purification

#### 2.7.1. Prokaryotes

REG was transformed into *E. coli* BL21(DE3) competent cells. A single colony was cultured in Luria broth medium with kanamycin at 37 °C until the OD_600_ attained 0.6–0.8. Protein expression was induced with 0.5 mmol/L IPTG, followed by shaking incubation at 37 °C for 6 h. Then, the culture was centrifuged at 8000 rpm for 10 min at 4 °C. The pellet was resuspended in ice-cold PBS and lysed via ultrasonication (300 W, 5 s on/5 s off, total 6 min). The lysate was centrifuged at 12,000 rpm for 20 min at 4 °C, and the supernatant was loaded onto a 20 mL Ni Elite Beads (TED) gravity-flow column pre-equilibrated with binding buffer. The column was washed with 10 column volumes of washing buffer (40 mM HEPES, 150 mM NaCl, 40 mM imidazole) to remove non-specific binding proteins. The target protein was next stepwise eluted with elution buffer (40 mM HEPES, 150 mM NaCl, 500 mM imidazole) at 1 mL per fraction. Eluates containing the target protein were pooled, concentrated using an ultrafiltration tube, and buffer-exchanged into PBS.

#### 2.7.2. Eukaryotes

The eukaryotic expression constructs encoding the HMPV G protein ectodomain (RMG) and the fusion proteins thereof (RMG-CRM197, RMG-CTB) were constructed as described in Section 2.5. All plasmids were co-transfected into 293F cells using polyethyleneimine (PEI) at a DNA:PEI mass ratio of 1:4 μg. After suspension culture at 37 °C under 5% CO_2_ for 5 days, the cells were centrifuged and the culture supernatants collected. Recombinant proteins were purified from the supernatants as described in Section 2.7 above.

### 2.8. Thermal Stability Analysis by Uncle

Hermal unfolding profiles of the recombinant proteins were analyzed using a Protein Stability Analysis System (UNcle, Unchained Labs, Pleasanton, CA, USA). Purified proteins were diluted to 0.5 mg/mL in PBS (pH 7.4). Aliquots of 9 μL were loaded into UNi capillary cassettes for measurement. Thermal scanning was performed from 15 °C to 95 °C at a heating rate of 0.8 °C/min. Intrinsic fluorescence intensity and barycentric mean (BCM) were continuously monitored throughout the temperature ramp. Protein melting behavior was evaluated according to the BCM shift as a function of temperature. All measurements were carried out in triplicate.

### 2.9. ELISA of IgG

Purified RMG protein was coated onto a 96-well ELISA plate (100 ng/well) using Beijing Solarbio Science & Technology Co., Ltd. (Beijing, China) ELISA Coating Buffer (1×, pH ~9.6, Cat. No. C1050) and incubated overnight at 4 °C. The plate was then blocked with 5% skimmed milk at 37 °C for 1 h. Diluted serum samples (100 μL/well) were next added, with an anti-His antibody (Proteintech Group, Inc., Rosemont, IL, USA, [Cat. No. 66005-1-Ig] ) serving as a standard, after serial dilution. Incubation at 37 °C for 1 h followed. After washing, horseradish peroxidase (HRP)-labeled goat anti-mouse IgG secondary antibody (1:1000 dilution, 100 μL per well) was added and incubation at 37 °C continued for 1 h. Samples were then incubated with the 3,3’,5,5’-tetramethylbenzidine chromogenic substrate in the dark for 15 min, and the color that developed was quantitated via optical density (OD) measurement at 450 nm. This yielded the IgG concentrations.

To verify the native antigenicity and correct folding of recombinant proteins, an additional indirect ELISA was performed using sera from HMPV-infected mice. Purified recombinant proteins (REG, RMG, G-CTB, G-CRM197) were coated and incubated overnight at 4 °C. After blocking, serially diluted infected mouse sera and naive mouse sera (negative control) were incubated for 1 h at 37 °C. HRP-conjugated goat anti-mouse IgG was used as the secondary antibody. Color development and OD450 measurement were performed as described above.

### 2.10. Mouse Serum Neutralization

Vero E6 cells were purchased from Procell Life Science & Technology Co. Ltd. ((Wuhan, China; Cat. No. CL-0491). Logarithmic-phase cells were digested, counted, and adjusted to 1 × 10^5^ cells/mL in fresh medium containing 2% FBS. The cell suspension was seeded into 96-well plates and incubated overnight.

Virus stocks of both HMPV lineage A (AY297749, recombinant GFP-expressing strain) and lineage B (AY525843) were diluted in serum-free DMEM to a working concentration corresponding to TCID50 = 200. Mouse serum samples were heat-inactivated at 56 °C for 30 min prior to use. Mouse serum was first diluted 25-fold using virus diluent, and then serially diluted 3-fold. And the virus-serum mixtures were incubated at 37 °C for 1 h. Following incubation, 100 μL of each mixture was transferred in sextuplicate to the pre-seeded Vero E6 cell monolayers. Meanwhile, serum-free culture medium containing only the virus was set up as a virus control, and wells containing only serum-free culture medium were set up as cell controls.

Plates were incubated at 37 °C with 5% CO_2_ for 48 h. For the GFP-expressing lineage A virus, fluorescence intensity was quantified using the Operetta CLS™ High-Content Analysis System. For lineage B virus, cytopathic effect (CPE) was observed microscopically.

No detectable or consistent neutralizing antibody titers (NT_50_) were observed against either HMPV lineage A or lineage B in any immunized group.

### 2.11. SDS-PAGE and Western Blot Analysis

Proteins were mixed with 5× SDS-PAGE loading buffer and denatured at 100 °C for 10 min. Electrophoresis was performed on a 4–20% precast gradient gel. Proteins were transferred onto a polyvinylidene fluoride membrane using the sandwich method. The membrane was blocked with Tris-buffered saline/Tween 20 (TBST) containing 5% skimmed milk for 1 h at room temperature, followed by overnight incubation at 4 °C with a mouse anti-His tag primary antibody. After washing with TBST, the membrane was incubated with an HRP-conjugated goat anti-mouse IgG secondary antibody for 1 h at room temperature. Following additional TBST washes, protein bands were visualized using electrochemiluminescent reagents mixed at a 1:1 ratio, and a chemiluminescence imaging system.

### 2.12. Statistical Analysis

Data analysis was performed using GraphPad Prism 10. The results are presented as medians (ranges) with the means ± standard deviations (SD). For comparisons involving more than two groups, one-significant difference (HSD) post hoc test for multiple pairwise comparisons. For comparisons between only two groups, the unpaired two-tailed Student’s t-test was used. A *p*-value < 0.05 after Tukey’s correction was considered statistically significant. A *p*-value < 0.05 was considered statistically significant (* *p* < 0.05, ** *p* < 0.01, *** *p* < 0.001).

## 3. Results

### 3.1. Expression and Confirmation of Recombinant Protein G Integrity

Given the weak immunogenicity of protein G, we fused it to CTB, an efficient antigen delivery vehicle. In addition, as native protein G is highly glycosylated, we fused it to CRM197, a carrier protein exhibiting high compatibility in terms of structure and function.

**Figure 2:**
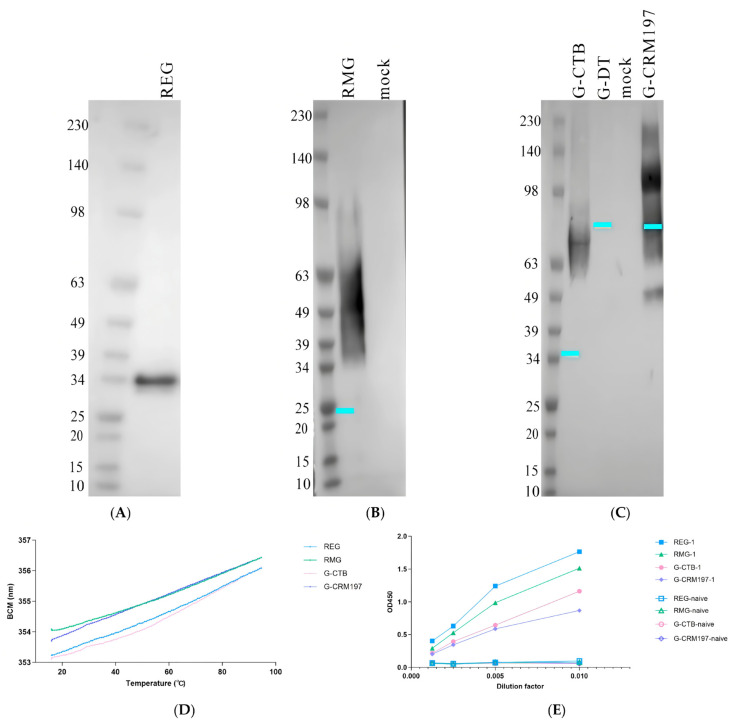
Characterization of recombinant HMPV G proteins: (**A**–**C**) Identification of the HMPV G protein and recombinants thereof via Western blotting. Expression of HMPV protein G and the recombinant derivatives is detected via anti-His tag Western blotting: (**A**) Prokaryotically expressed HMPV G protein (REG). (**B**) Eukaryotically expressed HMPV G protein (RMG). (**C**) HMPV G fused to carrier proteins (CTB/CRM197/DT); “mock” refers to the empty control for the corresponding expression system. Protein molecular weight standards (kDa) are shown on the left. The theoretical molecular weights of the proteins are calculated from their full amino acid sequences: 24.7 kDa for REG/RMG, 34.9 kDa for G-CTB, 81.7 kDa for G-DT, and 79.6 kDa for G-CRM197 (the cyan lines in the figures indicate positions corresponding to the theoretical molecular weights). The observed molecular weights are attributable to aberrant SDS-PAGE migration caused by the high serine, threonine, and proline contents of the G protein domain, even in the absence of glycosylation. (**D**) The barycentric mean (BCM) wavelength of intrinsic fluorescence as a function of temperature (15–95 °C) for REG, RMG, G-CTB, and G-CRM197. (**E**) Indirect ELISA reactivity of recombinant proteins with sera from HMPV-infected mice.

The expression product of the recombinant HMPV protein G was separated via SDS-PAGE electrophoresis and identified by Western blotting analysis using an anti-His tag antibody (Figure 2). For the prokaryotic expression product (REG), we observed a prominent uniform band at approximately 34 kDa (Figure 2A), which is much larger than the theoretical molecular weight of 24.7 kDa. Such anomalous electrophoretic migration is a well-documented characteristic of the HMPV G protein. The REG expressed in *E. coli* is not glycosylated but, rather, exhibits an extended, intrinsically disordered conformation given the high contents of serine, threonine, and proline residues. This conformation strongly impairs uniform SDS binding along the polypeptide chain and increases hydrodynamic resistance during protein migration in a gel matrix, ultimately creating Western blot bands at positions much higher than the theoretical molecular weight [22].

For the eukaryotically expressed protein RMG, we observed a diffuse band covering a molecular weight range of 35 to 98 kDa (Figure 2B), but without any specific band in the empty vector control lane. Diffuse banding is a characteristic feature of glycosylated proteins, suggesting that RMG underwent post-translational glycosylation in eukaryotic cells.

Turning to the recombinant proteins (G-CRM197, G-CTB, G-DT) we found that G-CTB and G-CRM197 exhibited specific bands at 50–90 kDa and 49–190 kDa, respectively (Figure 2C). Their theoretical molecular weights are 34.9 kDa and 81.7 kD. The banding patterns again reflected the characteristics of glycosylation, and indicated that the two carrier proteins were successfully fused to the G protein. Conversely, no specific band corresponding to G-DT was detected (Figure 2C), indicating that this construct was not effectively expressed in the eukaryotic system. These results confirm successful expression of the recombinant proteins G (REG, RMG, G-CRM197, G-CTB) in both the prokaryotic and eukaryotic systems, but the G-DT variant was not expressed.

Notably, the extracellular domain of G functions as a heavily glycosylated, intrinsically disordered polymer (IDP) characterized predominantly by random curls and flexible loops conformations, with no stable secondary or tertiary structures [23]. Thermal stability was assessed using an Uncle instrument across a temperature range of 15–95 °C. The barycentric mean (BCM) fluorescence wavelength shift as a function of temperature is presented in Figure 2D. In contrast to typical globular proteins, which undergo sharp, cooperative unfolding transitions, all four recombinant proteins displayed a gradual, near-linear increase in BCM signal with increasing temperature, with no discernible melting temperature (Tm). This behavior is fully consistent with the hallmark properties of IDPs, which undergo progressive structural expansion rather than cooperative folding/unfolding transitions.

To verify the antigenic authenticity of the recombinant proteins, an indirect ELISA was performed using sera from HMPV-infected mice. Serial dilutions of infected and naive mouse sera were incubated with the purified recombinant proteins. As shown in Figure 2E, all proteins (REG, RMG, G-CRM197, and G-CTB) were recognized by infected mouse sera in a clear dose-dependent manner, while naive sera exhibited no obvious binding. These results confirm that the recombinant proteins display native antigenic epitopes.

### 3.2. Recombinants of G with Carrier Proteins Induce a Stronger IgG Antibody Response

**Figure 3:**
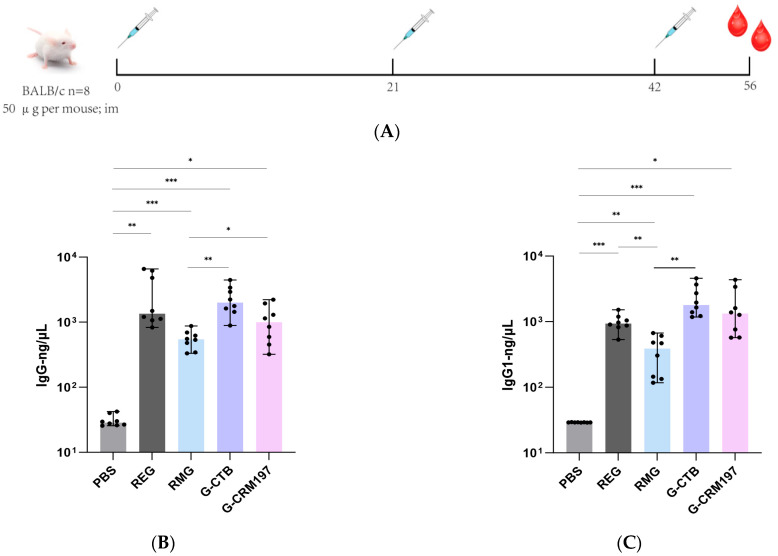

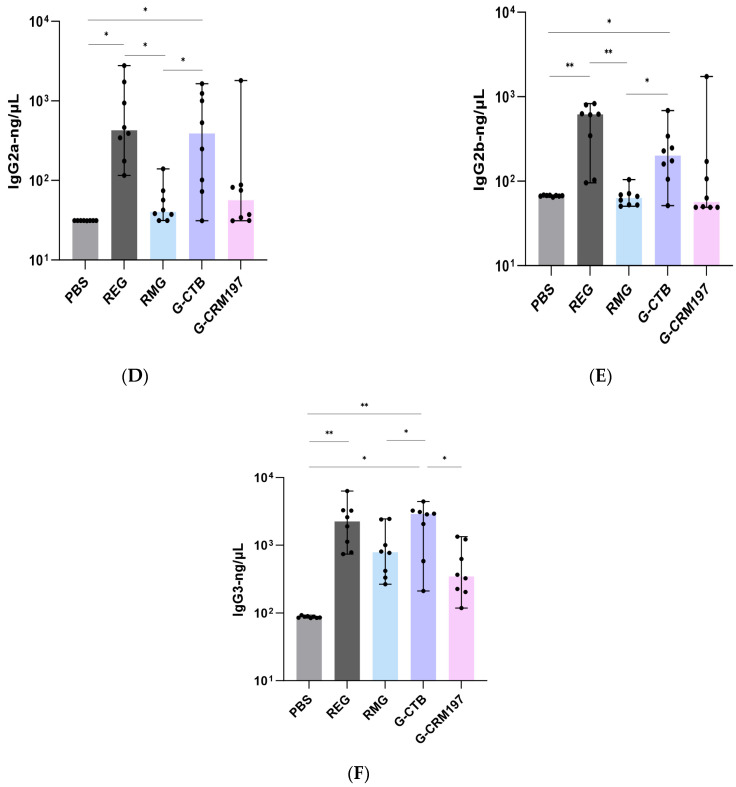
Total IgG antibody titers, IgG subclass distributions against HMPV A2 induced by HMPV G protein and recombinant constructs thereof. BALB/c mice (*n* = 8) are immunized via intramuscular injection on days 0, 21, and 42. On day 56, sera are collected and analyzed (by ELISA) in terms of specific antibody titers against HMPV G protein: IgG1, IgG2a, IgG2b, and IgG3 (*n* = 8). (**A**) Immunization schedule of BALB/c mice. (**B**) Total IgG antibody titer for G protein. (**C**) IgG1 antibody titer for G protein. (**D**) IgG2a antibody titer for G protein. (**E**) IgG2b antibody titer for G protein. (**F**) IgG3 antibody titer for G protein. Data are expressed as means ± SDs; statistical significance is indicated as follows: * *p* < 0.05, ** *p* < 0.01, *** *p* < 0.001.

To elucidate humoral immune responses, we measured the levels of total IgG and the subclasses thereof in sera from mice inoculated with HMPV G and the recombinant proteins G-CTB and G-CRM197 (Figure 3) using the three immunization protocols depicted in Figure 3A.

As shown in Figure 3B, REG induced significantly more antibody than did RMG. The PBS control group exhibited the lowest IgG concentration (<10^2^ ng/μL), consistent with the lack of specific antigenic stimulation. In contrast, REG elicited a relatively strong IgG response (>10^3^ ng/μL), significantly higher than that of the PBS control (*p* < 0.01). However, RMG induced a lower IgG concentration than did REG (<10^3^ ng/μL), suggesting that the post-translational glycosylation modifications of RMG may temporarily decrease the recognition efficiency of specific B-cell epitopes that may be masked by glycan chains. The recombinant G-carrier proteins (G-CTB, G-CRM197) induced total IgG concentrations that were significantly higher than those of RMG (*p* < 0.01, *p* < 0.05), highlighting the potent immunostimulatory effects of the carrier moieties.

As shown in Figure 3C,D, RMG induced a higher proportion of IgG1/IgG2a than did REG. In addition, compared with RMG, G-CTB elicited a significantly higher proportion of IgG2a/IgG1. The above results indicate that recombinant HMPV G, when combined with carrier proteins, particularly G-CTB, can induce higher levels of G-specific total IgG antibodies. These findings demonstrate that G-CTB modification shifts the antibody subclass distribution induced by the G protein closer to the unmodified state, thus avoiding the subclass distribution bias introduced by eukaryotically expressed RMG.

### 3.3. Protective Effects of Recombinant G Proteins Against Viral Challenge in Mice and Assessment of Pulmonary Inflammation

**Figure 4:**
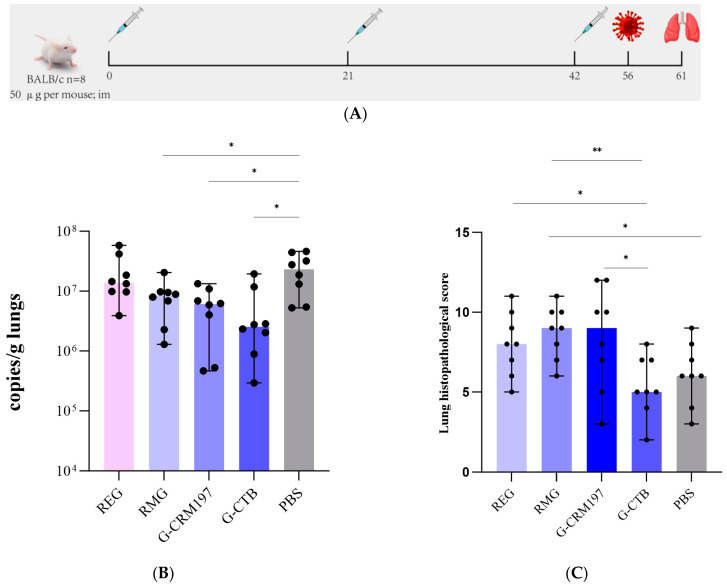

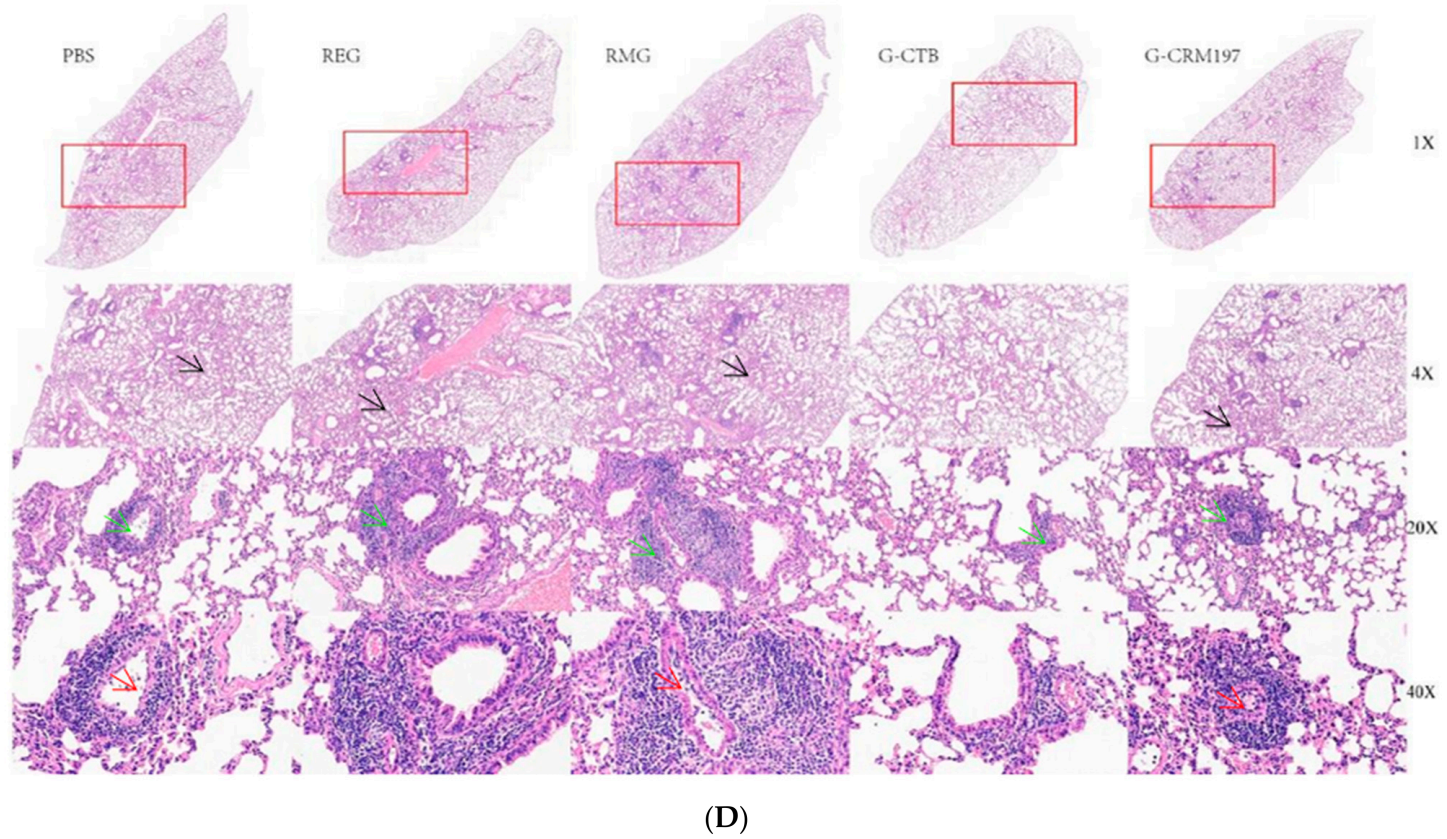
Protective efficacy of HMPV G and recombinants thereof against HMPV challenge, and risk assessment of lung inflammation in BALB/c mice: (**A**) BALB/c mice (*n* = 8) are immunized via intramuscular injection on days 0, 21, and 42. On day 56, they are inoculated intranasally with HMPV at a dose of 1 × 10^7^ PFU. Lung tissues are harvested on Day 61. (**B**) Viral copies/g lung tissue on day 5 post-challenge. (**C**) Pulmonary histopathology scores. (**D**) Representative H&E-stained lung tissue sections (magnifications: 1×, 4×, 20×, 40×). Black arrows indicate thickened alveolar septa; green arrows peribronchial lymphocytic infiltrations, and red arrows bronchial lumen exudations. Data are expressed as means ± SDs; statistical significance is indicated as follows: * *p* < 0.05, ** *p* < 0.01.

We next performed an HMPV challenge experiment (Figure 4A). As shown in Figure 4B, viral loads in lung tissues were measured on day 5 post-challenge. Compared with the PBS control group, viral titers were reduced in the RMG, G-CTB, and G-CRM197 groups (*p* < 0.05), suggesting that RMG and the two recombinant proteins triggered viral clearance by mice. Notably, although the viral titers in the RMG group were significantly lower than those of the PBS group (*p* < 0.05), this reduction was markedly weaker than that observed for G-CTB. REG did not reduce the viral load. The histopathological scores and the H&E-stained lung sections obtained on day 5 post-challenge are shown in Figure 4C,D. Slides were scored blindly by two independent pathologists. Alveolar septal thickening, a hallmark of interstitial inflammation and edema, was more severe in the RMG than the REG group, suggesting that the glycosylated HMPV G protein may induce more lung injury. In contrast, the pathological scores were significantly lower in the G-CTB than in both the RMG and REG groups. As shown in Figure 4D (magnifications: 1×, 4×, 20×, 40×), the severity of inflammation was markedly reduced in the G-CTB group compared with the other groups. At low magnification (1×), alveolar septal thickening was substantially milder in the G-CTB group than other groups, reflecting reduced interstitial inflammation and less pulmonary edema. At high magnification (20–40×), peribronchial inflammatory infiltrations were observed in all groups, and the infiltrating cells were morphologically identified as (predominantly) lymphocytes based on the characteristic small round nuclei and scanty cytoplasm. Only mild or no lymphocytic infiltration was observed in the G-CTB group, whereas the RMG group exhibited the most severe inflammatory changes, characterized by dense, circumferential peribronchial lymphocytic infiltrations more than 10 cell layers in thickness. Among all groups, the RMG and G-CRM197 groups evidenced the highest overall inflammatory burdens, whereas the G-CTB group exhibited the mildest lung inflammation.

Together, our findings indicate that immunization using HMPV G alone modestly reduced the viral load, but enhanced the immunopathology. However, when G was fused to CTB, the immunogenicity increased, the lung viral load fell, and severe lung inflammation was significantly attenuated during virus challenge.

## 4. Discussion

Given that extensive glycosylation of the G protein may contribute to low immunogenicity, this study aimed to enhance immunogenicity via fusion of G with CRM197 and CTB, based on the fundamental design principle of polysaccharide conjugate vaccines. CRM197 is the most widely used and well-established carrier protein in licensed conjugate vaccines, and exhibits high structural and functional compatibility. Derived from diphtheria toxin, CRM197 carries a single key point mutation (G52E) that abolishes ADP-ribosyltransferase activity and thereby renders the protein completely non-toxic [20] while preserving the conformation of the receptor-binding domain. CRM197 thus retains efficient binding to proheparin-binding epidermal growth factor-like growth factor (proHB-EGF), which is broadly expressed on human cell surfaces [24]. When conjugated to a polysaccharide antigen, the CRM197 complex is efficiently internalized by antigen-presenting cells via receptor-mediated endocytosis. The CRM197 moiety is then processed into peptides and presented to CD4^+^ T helper cells via MHC class II molecules. Activated T-cells deliver costimulatory signals to B-cells, converting weakly T-cell-independent polysaccharide antigens into strong T-cell-dependent antigens that induce high-affinity IgG antibodies, immune memory, and class switching. The safety and efficacy of CRM197 have been fully validated, and the material is used in many licensed vaccines against *Hemophilus influenzae* type b, pneumococcus, and meningococcus [24].

CTB is the nontoxic B-subunit of cholera toxin, which self-assembles from five identical 11.6- kDa monomers into a highly stable, cyclic pentameric structure [25]. When weakly immunogenic glycoproteins are coupled to CTB via genetic fusion or chemically, CTB serves as an efficient antigen delivery vehicle and immune activation platform, transforming weakly immunogenic glycoproteins that are poorly recognized by the immune system into effective antigens capable of eliciting strong and durable immune responses [26].

REG induced higher total IgG levels than did RMG, supporting the notion that dense eukaryotic glycosylation may shield B-cell epitopes and reduce immunogenicity. Conversely, G-CTB and G-CRM197 elicited markedly higher IgG responses than did RMG, confirming that carrier proteins enhance antigen presentation and B-cell activation, thereby overcoming the immunogenicity limitations imposed by heavy glycosylation. A critical safety concern with pneumovirus G proteins is their potential to drive Th2-skewed immune responses, which are closely linked to VED [27]. In the study, RMG induced a higher proportion of IgG1 (a Th2-associated antibody subclass) and more severe lung inflammation than did REG, suggesting that glycosylation may be associated with a shift toward a Th2-biased humoral response induced by the G protein. Encouragingly, fusion to CTB strongly increased the proportion of IgG2a (a Th1-associated antibody subclass), indicating a potential modulation of the antibody subclass distribution. These data suggest that CTB may not only enhance G immunogenicity but also redirect the humoral immune profile away from an excessive Th2-like pattern, which in turn may contribute to reduced Th2-driven lung inflammation and pathology. From a translational perspective, this represents a promising strategy to mitigate VED-like risks by modulating humoral immune profiles using carrier proteins. It should be emphasized that formal T-cell phenotyping, antigen-specific stimulation assays, and cytokine profiling were not conducted in the present study. Accordingly, any conclusions pertaining to direct Th1/Th2 immune balance remain inferential. In future work, we will further explore the underlying mechanisms by implementing antigen-specific T-cell stimulation and cytokine profiling analyses, so as to directly validate the impacts of our vaccine candidates on Th1/Th2 immune homeostasis.

In the present study, no recombinant G protein induced neutralizing antibodies, consistent with the findings of previous reports to the effect that HMPV protein G does not elicit, or only weakly elicits, a protective response when administered directly as an immunogen [28]. This may be due to occlusion of neutralizing epitopes by glycosylation and the inability of the ectodomain to recapitulate native conformational neutralizing epitopes. Despite the absence of neutralizing antibodies, G-CTB and G-CRM197 still promoted viral clearance in mice, suggesting the involvement of non-neutralizing antibody-dependent cellular protective mechanisms that warrant further investigation. Although the observed reduction in viral load was modest and did not reach the protective potency of pre-F-based vaccines [29], the major goal of this study was not to develop a standalone protective vaccine, but to determine whether the poorly immunogenic HMPV G protein could be converted into a safe and immunogenic antigen via carrier protein fusion. Importantly, unmodified RMG not only showed weak protection but also exacerbated lung inflammation compared with the PBS control group, underscoring the safety risks of native G-based immunogens. In contrast, G-CTB significantly improved specific IgG responses, reduced lung viral load, and alleviated pulmonary pathology relative to RMG. Thus, the key advance of this work is the successful conversion of a weakly immunogenic, potentially pathogenic antigen into a safe and immunogenic vaccine component.

Despite these promising findings, the present study has several limitations that should be acknowledged.

First, comprehensive immunological characterization and long-term durability of immune responses were not assessed. Although we evaluated G-specific IgG levels and antibody subclass distribution, this study did not examine memory B-cell formation, T-memory cell subsets, or the persistence of antibody responses over time. As protective vaccine immunity depends not only on response magnitude but also on immune durability, these gaps limit the full understanding of the clinical potential of G-based antigens. In addition, since pre-F protein is the major target for potent neutralizing antibodies against HMPV, the lack of neutralizing activity observed here further highlights the complementary role rather than a replacement role of G-based antigens in future vaccine design.

Second, homologous challenge with lineage B virus would be ideal for evaluating lineage-specific protective efficacy. However, we were unable to propagate lineage B virus to a sufficiently high titer for mouse challenge despite repeated attempts. Our cross-lineage challenge design may therefore slightly underestimate protection against homologous lineage B infection. Nevertheless, this limitation does not affect our core conclusions. Neutralization assays against both lineages confirmed that G-based immunization failed to induce detectable neutralizing activity against either lineage, indicating that this is an intrinsic property of G antigen rather than a lineage-specific effect. Moreover, HMPV lineages A and B are antigenically related and co-circulate globally, so cross-lineage protection has practical translational value [30]. Once a high-titer lineage B challenge virus becomes available, homologous challenge studies should be performed to further validate protective efficacy.

Collectively, our findings support the suggestion that HMPV G is a viable candidate antigen for an HMPV vaccine, and we offer a feasible approach toward improvement of immunogenicity and safety.

## Data Availability

Data are available upon reasonable request. All data are available in the main text or Supplementary Materials. Correspondence and requests for materials should be addressed to Jizhen Chen and Changgui Li. Data are available upon reasonable request. All data are available in the main text or Supplementary Materials.

## Supplementary Materials

The following supporting information can be downloaded at: https://www.mdpi.com/article/10.3390/tropicalmed11050135/s1, Table S1: Amino acid sequences and NCBI RefSeq accession numbers of the recombinant constructs. The table lists the full amino acid sequence and corresponding NCBI RefSeq accession number for each construct used in this study.

## References

[1] Shafagati N., Williams J. (2018). Human metapneumovirus—What we know now. F1000Research.

[2] Wise J. (2023). HMPV: The little known virus that’s making its mark in intensive care. BMJ.

[3] Chittiprol N., Kandi V., Pinnelli V.B.K., Suvvari T.K., Madamsetti N., Ca J., Challa S.T. (2025). The Re-emergence of Human Metapneumovirus: Virus Classification, Characteristics, Mechanisms of Infection, Clinical Features, Diagnosis, Epidemiology, Prevention, and Treatment. Cureus.

[4] van den Hoogen B.G., de Jong J.C., Groen J., Kuiken T., de Groot R., Fouchier R.A., Osterhaus A.D. (2001). A newly discovered human pneumovirus isolated from young children with respiratory tract disease. Nat. Med..

[5] Sah R., Srivastava S., Kumar S., Koteswara Rao G.S.N., Mehta R., Mohanty A., Sah S., Mehta V., Feehan J., Apostolopoulos V. (2025). Ongoing hMPV outbreaks in China and other Asian countries. Infez. Med..

[6] Meher M.M., Afrin M. (2026). Global surge of human metapneumovirus (hMPV) and its interactions with microbiome to disease severity. J. Infect. Public Health.

[7] Principi N., Fainardi V., Esposito S. (2025). Human Metapneumovirus: A Narrative Review on Emerging Strategies for Prevention and Treatment. Viruses.

[8] Schuster J.E., Williams J.V. (2014). Human Metapneumovirus. Microbiol. Spectr..

[9] Tiruthani K., Card M.P., Wolf W., Shen L., Schaefer A., Fritz M., Okuda K., Harkema J.R., Mousa J.J., R J.P. (2025). Preferential apical infection and spread of human metapneumovirus highlights the importance of inhaled delivery of neutralizing monoclonal antibody to treat established infections. Proc. Natl. Acad. Sci. USA.

[10] Sharma R., Walia A., Lakhanpal D. (2026). Human metapneumovirus: An underdiagnosed public health threat. Infect. Dis. Now..

[11] Kumar P., Srivastava M. (2018). Prophylactic and therapeutic approaches for human metapneumovirus. Virusdisease.

[12] Kahn J.S. (2006). Epidemiology of human metapneumovirus. Clin. Microbiol. Rev..

[13] Velayutham T.S., Ivanciuc T., Garofalo R.P., Casola A. (2022). Role of human metapneumovirus glycoprotein G in modulation of immune responses. Front. Immunol..

[14] Thammawat S., Sadlon T.A., Hallsworth P.G., Gordon D.L. (2008). Role of cellular glycosaminoglycans and charged regions of viral G protein in human metapneumovirus infection. J. Virol..

[15] Eagle A. (2012). Driven design. Process improvement tools guide replacement project. Health Facil. Manag..

[16] Dubois J., Pizzorno A., Cavanagh M.H., Padey B., Nicolas de Lamballerie C., Uyar O., Venable M.C., Carbonneau J., Traversier A., Julien T. (2019). Strain-Dependent Impact of G and SH Deletions Provide New Insights for Live-Attenuated HMPV Vaccine Development. Vaccines.

[17] Kolli D., Bao X., Liu T., Hong C., Wang T., Garofalo R.P., Casola A. (2011). Human metapneumovirus glycoprotein G inhibits TLR4-dependent signaling in monocyte-derived dendritic cells. J. Immunol..

[18] Liu L., Bastien N., Li Y. (2007). Intracellular processing, glycosylation, and cell surface expression of human metapneumovirus attachment glycoprotein. J. Virol..

[19] van der Put R.M.F., Metz B., Pieters R.J. (2023). Carriers and Antigens: New Developments in Glycoconjugate Vaccines. Vaccines.

[20] Malito E., Bursulaya B., Chen C., Lo Surdo P., Picchianti M., Balducci E., Biancucci M., Brock A., Berti F., Bottomley M.J. (2012). Structural basis for lack of toxicity of the diphtheria toxin mutant CRM197. Proc. Natl. Acad. Sci. USA.

[21] Salguero F.J., White A.D., Slack G.S., Fotheringham S.A., Bewley K.R., Gooch K.E., Longet S., Humphries H.E., Watson R.J., Hunter L. (2021). Comparison of rhesus and cynomolgus macaques as an infection model for COVID-19. Nat. Commun..

[22] Ryder A.B., Tollefson S.J., Podsiad A.B., Johnson J.E., Williams J.V. (2010). Soluble recombinant human metapneumovirus G protein is immunogenic but not protective. Vaccine.

[23] Leyrat C., Paesen G.C., Charleston J., Renner M., Grimes J.M. (2014). Structural insights into the human metapneumovirus glycoprotein ectodomain. J. Virol..

[24] Broker M., Costantino P., DeTora L., McIntosh E.D., Rappuoli R. (2011). Biochemical and biological characteristics of cross-reacting material 197 CRM197, a non-toxic mutant of diphtheria toxin: Use as a conjugation protein in vaccines and other potential clinical applications. Biologicals.

[25] Baldauf K.J., Royal J.M., Hamorsky K.T., Matoba N. (2015). Cholera toxin B: One subunit with many pharmaceutical applications. Toxins.

[26] Royal J.M., Matoba N. (2017). Therapeutic Potential of Cholera Toxin B Subunit for the Treatment of Inflammatory Diseases of the Mucosa. Toxins.

[27] Bao X., Kolli D., Ren J., Liu T., Garofalo R.P., Casola A. (2013). Human metapneumovirus glycoprotein G disrupts mitochondrial signaling in airway epithelial cells. PLoS ONE.

[28] Skiadopoulos M.H., Biacchesi S., Buchholz U.J., Amaro-Carambot E., Surman S.R., Collins P.L., Murphy B.R. (2006). Individual contributions of the human metapneumovirus F, G, and SH surface glycoproteins to the induction of neutralizing antibodies and protective immunity. Virology.

[29] Li H., Zhang J., Wang K., Bernardini S., Zhang H., Luo Y. (2025). Human metapneumovirus prevention within reach: Advances in vaccines and monoclonal antibodies. Lancet Microbe.

[30] Skiadopoulos M.H., Biacchesi S., Buchholz U.J., Riggs J.M., Surman S.R., Amaro-Carambot E., McAuliffe J.M., Elkins W.R., St Claire M., Collins P.L. (2004). The two major human metapneumovirus genetic lineages are highly related antigenically, and the fusion (F) protein is a major contributor to this antigenic relatedness. J. Virol..

