# Supplementary material for: Human Metapneumovirus G Protein Immunogenicity and Safety Explored via Carrier Protein Fusion
Source: Trop Med Infect Dis. 2026 May 15;11(5):135. doi: 10.3390/tropicalmed11050135 (PMC13211399; doi:10.3390/tropicalmed11050135)
Supplement: Supplementary file 1 [file tropicalmed-11-00135-s001.zip › tropicalmed-4235715-supplementary.pdf]

Supplementary Table 1 The sequences of the proteins

Protein<br>(NCBI RefSeq)	SEQUENCE（N’-C’）
HMPV G<br>（PP086008.1）	TMQENTSESEHHTSSSPMESSRETPTVPIDNSDTNPSSQ<br>YPTQQSTEGSTLYFAASASSPETEPTSTPDTTSRPPFVDT<br>HTTPPSASRTKTSPAVHTKNNPRISSRTHSPPWAMTRTV<br>RRTTTLRTSSIRKRSSTASVQPDSSATTHKHEEASPVSPQ<br>TSASTMRGQHINNIQPN
DT<br>（WP_029975703.1）	MNRKLFASILIGALLGIGAPPSAHAGADDVVDSSKSFV<br>MENFSSYHGTKPGYVDSIQKGIQKPKSGTQGNYDDDW<br>KGFYSTDNKYDAAGYSVDNENPLSGKAGGVVKVTYP<br>GLTKILALKVDNAETIKKELGLSLTEPLMEQVGTEEFIK<br>RFGDGASRVVLSLPFAEGSSSVEYINNWEQAKELSVEL<br>EINFETRGKRGQDAMYEYMAQSCAGNRVRRSVGSSLS<br>CINLDWDAIRDKTKTKIESLKEHGPIKNKMSESPNKTV<br>SEEKAKQYLEEFHRTALEHPELSELKTVTGVNSVFAGA<br>NYASWAVNVAQVVDSKTADNLEKTTAALSILPGIGSVM<br>GIADGAVHHNTEEIVAQSIALSSLMVAQAIPLVGELVDL<br>GFAAYNFVESIINLFQVVHNSYNRPAYSPGHKTQPFVH<br>GGYAASWNTVEDSIIKTGFQGESGHDIKITAENTPLPIA<br>GVLLPTIPGKLDVNKSKTHISVNGRKIRMRCRAIDDATT<br>FCRPKTPVYVGNGVHANLHVAFHTSSSEKIHSDETPLSS<br>IDVLGYQKTVDHTKVNSKLSLFAEVKS
CTB<br>（WP_000593523.1）	MIKLKFGVFFTVLLSSAYANGTPQNITDLCAEYHNTQIH<br>TLNDKIFSYTESLAGKREMAIITFKNGATFQVEVPGSQH<br>IDSQKKAIERMKDTLRIAYLTEAKVEKLCVWNNKTPH<br>AIAAISMAN
CRM197<br>（AMV91693.1）	MGADDVVDSSKSFVMENFSSYHGTKPGYVDSIQKGIQ<br>KPKSGTQGNYDDDWKEFYSTDNKYDAAGYSVDNENP<br>LSGKAGGVVKVTYPGLTKVLALKVDNAETIKKELGLS<br>LTEPLMEQVGTEEFIKRFGDGASRVVLSLPFAEGSSSVE<br>YINNWEQAKALSVELEINFETRGKRGQDAMYEYMAQ<br>ACAGNRVRRSVGSSLSCINLDWDVIRDKTKTKIESLKE

HGPIKNKMSESPNKTVSEEKAKQYLEEFHQTALEHPEL
SELKTVTGTNPVFAGANYAAWAVNVAQVIDSETADNLE
KTTAALSILPGIGSVMGIADGAVHHNTEEIVAQSIALSSL
MVAQAIPLVGELVDIGFAAYNFVESIINLFQVVHNSYNR
PAYSPGHKTQPFLHDGYAVSWNTVEDSIIRTGFQGESGH
DIKITAENTPLPIAGVLLPTIPGKLDVNKSKTHISVNGRK
IRMRCRAIDGDVTFCRPKSPVYVGNGVHANLHVAFHR
SSSEKIHSNEISSDSIGVLGYQKTVDHTKVNSKLSLFFEI
KS
